# Crystal structure of the human carbonic anhydrase II adduct with 1-(4-sulfamoylphenyl-ethyl)-2,4,6-triphenylpyridinium perchlorate, a membrane-impermeant, isoform selective inhibitor

**DOI:** 10.1080/14756366.2017.1405263

**Published:** 2017-12-04

**Authors:** Vincenzo Alterio, Davide Esposito, Simona Maria Monti, Claudiu T. Supuran, Giuseppina De Simone

**Affiliations:** a Istituto di Biostrutture e Bioimagini-CNR, Naples, Italy;; b Neurofarba Department, Section of Pharmaceutical and Nutraceutical Sciences, Università degli Studi di Firenze, Sesto Fiorentino, Florence, Italy

**Keywords:** X-ray crystallography, carbonic anhydrase, membrane-impermeant inhibitors

## Abstract

Pyridinium containing sulfonamides have been largely investigated as carbonic anhydrase inhibitors (CAIs), showing interesting selectivity features. Nevertheless, only few structural studies are so far available on adducts that these compounds form with diverse CA isoforms. In this paper, we report the structural characterization of the adduct that a triphenylpyridinium derivative forms with hCA II, showing that the substitution of the pyridinium ring plays a key role in determining the conformation of the inhibitor in the active site and consequently the binding affinity to the enzyme. These findings open new perspectives on the basic structural requirements for designing sulfonamide CAIs with a selective inhibition profile.

## Graphical Abstract

**Figure:**
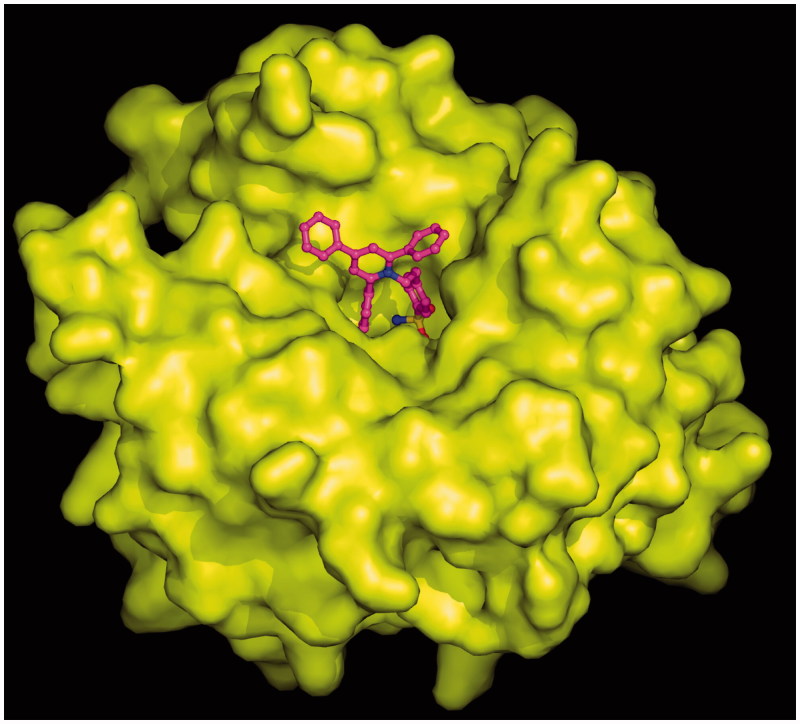

## Introduction

**Figure 1.**
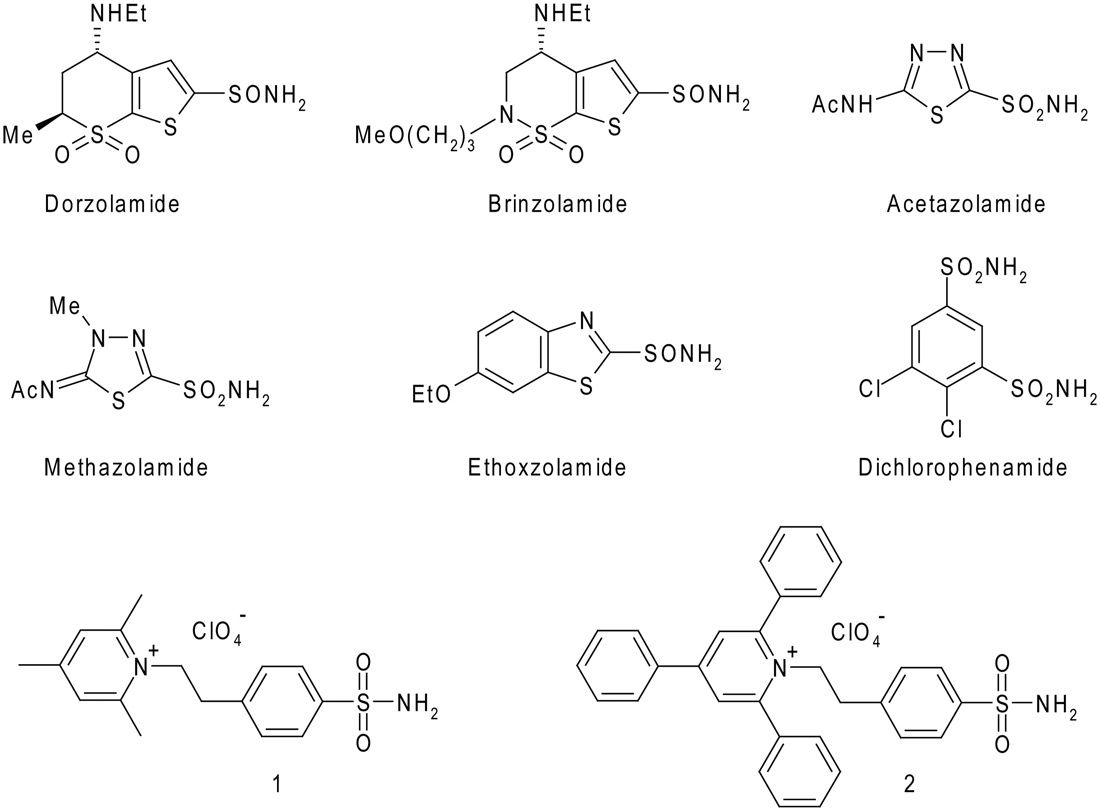
Chemical structures of clinically used CAIs and pyridinium containing sulfonamides **1** and **2**.

Heterocyclic/aromatic sulfonamides such as dorzolamide, brinzolamide, acetazolamide, methazolamide, ethoxzolamide, and dichlorophenamide (Figure 1) represent the first generations of clinically used inhibitors of the metalloenzyme carbonic anhydrase (CA) (EC 4.2.1.1)^1–3^. They are very strong, typically low nanomolar inhibitors, of most of the 15 CA isoforms presently known in humans^2^. Dorzolamide and brinzolamide are widely used, topically-acting antiglucoma agents^4^, ethoxzolamide^5^ has fewer clinical applications, whereas acetazolamide, methazolamide, and dichlorophenamide are systemically used antiglaucoma drugs, still employed clinically, even if they were discovered decades ago^4^. The latter compounds also show clinical benefits for the treatment of other conditions such as epilepsy^6–8^, idiopathic intracranial hypertension^9^, obesity^10–12^, and as diuretics^13^, but their applications are severely limited due to a range of side effects connected with inhibition of CA isoforms not involved in those specific pathologies^2^. For this reason, the development of isoform-selective CA inhibitors (CAIs) represented an important drug design challenge for the last two decades^2^^,^^14^, leading to interesting developments, with several classes of compounds identified so far, which show selective inhibition of the different CA isoforms. They belong to sulfonamide^15^, coumarin^16–18^, sulfocoumarin^19^^,^^20^, polyamine^21^, dithiocarbamate^22^^,^^23^, carboxylate^24^^,^^25^ chemotypes, among others. Such isoform-selective compounds opened new scenarios for the applications of CAIs as antitumor drugs^26–29^, anti-neuropathic pain agents^30^^,^^31^, or even for the management of cerebral ischemia^32^, arthritis^33^, or bacterial/fungal/protozoan infections^34^^,^^35^.

One of the most interesting class of isoform-selective sulfonamide CAIs is constituted by the pyridinium salts, obtained by reaction of amino-benzenesulfonamides with pyrylium salts^36–38^. These compounds represent the first class of CAIs, which were demonstrated to possess a high selectivity for inhibiting membrane-associated (CA IV, IX, XII, and XIV) over cytosolic or mitochondrial CA isoforms^36^^,^^37^^,^^39^. Furthermore, due to their cationic nature, they are also membrane-impermeant^36–38^, which makes them highly attractive for targeting extracellular CAs^26^^,^^39^^,^^40^. Among the pyridinium containing sulfonamides, compounds **1** and **2**, which incorporate 2,4,6-trisubstituted pyridinium moieties, were shown by our group to possess low nanomolar affinity for CA IX, a tumour-associated enzyme validated as an antitumor target^26^, and also to be less effective inhibitors of widespread, off target isoforms CA I, II, and IV^36^^,^^37^^,^^39^.

Despite the interesting selectivity features of the pyridinium containing sulfonamides as CAIs, only few structural studies are so far available on the adducts that these compounds form with diverse CA isoforms, with the hCA II/**1** complex being the only one characterized by X-ray diffraction studies^41^. In order to get more insights into the CA binding mechanism and the interesting selective inhibition profile of these molecules, we report here the crystal structure of the hCA II/**2** adduct and compare it with the previously described structure of the hCA II/**1** complex^41^. The inhibition of the second tumour-associated isoform, hCA XII, with derivatives **2** is also reported here for the first time.

## Materials and methods

### CA inhibition

Inhibition constants reported in Table 1 were previously determined^1^^,^^39^ with the exception of *K_I_* of **2** against hCA XII which has been determined here. In detail, an applied photophysics stopped-flow instrument has been used for assaying the CA catalyzed CO_2_ hydration activity^42^. Phenol red (at a concentration of 0.2 mM) has been used as indicator, working at the absorbance maximum of 557 nm, with 20 mM Hepes (pH 7.5) as buffer and 20 mM Na_2_SO_4_ (for maintaining constant the ionic strength), following the initial rates of the CA-catalyzed CO_2_ hydration reaction for a period of 10–100 s. The CO_2_ concentrations ranged from 1.7 to 17 mM for the determination of the kinetic parameters and inhibition constants. Six traces of the initial 5–10% of the reaction have been used for determining the initial velocity. The uncatalyzed rate was determined in the same manner and subtracted from the total observed rate. Stock solution of inhibitors (0.1 mM) were prepared in distilled–deionized water and dilutions up to 0.01 nM were done thereafter with the assay buffer. Inhibitor and enzyme solutions were preincubated together for 15 min at room temperature prior to assay, to allow for the formation of the *E*–*I* complex. The inhibition constants were obtained by non-linear least-squares methods using PRISM 3 (GraphPad Software Inc., San Diego, CA, USA) and the Cheng–Prusoff equation and represent the mean from at least three different determinations.

**Table 1.** Inhibition of isozymes hCA I, hCA II, hCA IV, hCA IX, and hCA XII with the pyridinium salts **1**, **2**, and the standard, clinically used sulfonamide CAIs.

	*K_*I*_* (nM)				
Compound	hCA I	hCA II	hCA IV	hCA IX	hCA XII
**1**	4000^a^	21^a^	60^a^	14^a^	7.0^a^
**2**	270,000^b^	419^b^	1830^b^^,^^d^	95^b^	12.5^c^
Dorzolamide	50,000^a^	9^a^	8500^a^	52^a^	3.5^a^
Brinzolamide	45,000^a^	3^a^	3950^a^	37^a^	3.0^a^
Acetazolamide	250^a^	12^a^	74^a^	25^a^	5.7^a^
Methazolamide	50^a^	14^a^	6200^a^	27^a^	3.4^a^
Ethoxzolamide	25^a^	8^a^	93^a^	34^a^	22^a^
Dichlorophenamide	1200^a^	38^a^	1500^a^	50^a^	50^a^

a From Ref. (1).

b From Ref. (39).

c This work.

d This *Ki* value refers to bCA IV.

### X-ray crystallography

The hCA II/**2** adduct was obtained using a procedure previously described for other hCA II/inhibitor complexes^43^^,^^44^. In detail, a 50-fold excess of the inhibitor was added to a 0.2 mg/mL enzyme solution in 20 mM Tris-HCl pH 8.0. After incubation overnight at 4 °C, the complex was concentrated to 10 mg/mL by using a 5-kDa cutoff ultrafiltration device (Vivaspin^®^ 500; Sartorius, Göttingen, Germany). Crystals were obtained at 20 °C using the hanging drop vapour diffusion technique by equilibrating drops containing 1 µL of complex solution and an equal volume of precipitant solution consisting of 1.3 M sodium citrate, 0.1 M TRIS-HCl, pH 8.5, over a reservoir containing 0.5 mL of precipitant solution. Crystals appeared after 3 days. Diffraction data were collected to 1.65 Å resolution, in-house at −180 °C, using a Rigaku MicroMax-007 HF generator producing Cu Kα radiation and equipped with a Saturn 944 CCD detector. Cryoprotection of the crystals was achieved transferring the crystals into the precipitant solution with the addition of 10% (v/v) glycerol. Data were indexed, integrated, and scaled using HKL2000^45^. Crystal parameters and data collection statistics are summarized in Table 2.

**Table 2.** Data collection and refinement statistics for the hCA II/**2** complex.

Crystal parameters	
Space group	*P2_*1*_*
*a* (Å)	42.1
*b* (Å)	41.3
*c* (Å)	71.9
*γ* (°)	104.2
Number of independent molecules	1
Data collection	
Resolution (Å)	25.3–1.65
Wavelength (Å)	1.54178
Temperature (K)	100
*R*-merge (%)^a^	5.9 (26.6)
<I>/<*σ*(*I*)>	25.7 (3.8)
Total reflections	172066
Unique reflections	27539
Redundancy	6.2 (2.7)
Completeness (%)	94.5 (79.6)
Refinement	
Resolution (Å)	25.3–1.65
*R*-work (%)^b^	17.5
*R*-free (%)^b^	21.0
RMSD from ideal geometry:	
Bond lengths (Å)	0.010
Bond angles (°)	1.6
Number of protein atoms	2076
Number of water molecules	195
Number of inhibitor atoms	36
Average *B* factor (Å^2^):	
All atoms	15.0
Protein atoms	14.2
Inhibitor atoms	26.5
Water molecules	22.2
Ramachandran statistics (%):	
Most favoured	88.2
Additionally allowed	11.4
Generously allowed	0.5
Disallowed	0

Values in parentheses are statistics for the highest resolution shell (1.68–1.65 Å).

a *R*-merge = Σ*_hkl_*Σ*_i_*|*I_i_*(*hkl*) − <*I*(*hkl*)>|/ Σ*_hkl_*Σ*_i_I_i_*(*hkl*), where *I_i_*(*hkl*) is the intensity of an observation and <*I*(*hkl*)> is the mean value for its unique reflection; summations are over all reflections.

b *R*-work = Σ*_hkl_*ǁ*F*_o_(*hkl*)| − |*F*_c_(*hkl*)ǁ/Σ*_hkl_*|*F*_o_(*hkl*)| calculated for the working set of reflections. *R*-free is calculated as for *R*-work, but from 4.9% of the data that was not used for refinement.

A previously solved structure of hCA II (PDB code 5O07)^46^, with inhibitor and non-protein atoms omitted, was used as starting model for rigid body refinement in CNS^47^^,^^48^. Initial refinement was continued in CNS using positional and slow cooling protocols followed by restrained *B*-value refinement. The inhibitor molecule was identified from peaks in |*F*_o_| − |*F*_c_| maps and gradually built into the model over several rounds of refinement. Composite simulated-annealing omit maps were used regularly during the building process to verify and correct the model^47^^,^^48^. Crystallographic refinement was carried out against 95.1% of the measured data. The remaining 4.9% of the observed data, which was randomly selected, was used for *R*-free calculations to monitor the progress of refinement. Topology files of the inhibitor were generated using the PRODRG2 server^49^. Restraints on inhibitor bond angles and distances were taken from similar structures in the Cambridge Structural Database^50^, whereas standard restraints were used on protein bond angles and distances throughout refinement. The correctness of stereochemistry was finally checked using PROCHECK^51^. The refinement statistics of final model are summarized in Table 2. Coordinates and structure factors were deposited in the Protein Data Bank (accession code 6EQU).

## Results and discussion

Sulfonamides **1** and **2** were previously described by our groups, and were obtained by reaction of 4-aminoethyl-benzenesulfonamide with 2,4,6-trisubstituted pyrylium salts^36^^,^^38^. In Table 1, the CA inhibitory action of these two positively-charged, membrane-impermeant sulfonamides, as well as those of the six clinically used drugs shown in Figure 1, are presented. From the table it is evident that acetazolamide, methazolamide and ethoxzolamide are promiscuous CAIs, inhibiting effectively at least four of the five investigated isoforms, whereas dichlorophenamide, dorzolamide, and brinzolamide possess a more selective inhibition profile, as their activity against hCA I and hCA IV are modest, being however effective inhibitors of three isoforms, hCA II, IX, and XII. A different inhibition profile is observed for the two pyridinium-containing sulfonamides. Indeed, whereas the trimethylpyridinium derivative **1** is a low nanomolar inhibitor of hCA IX and XII, effectively inhibits hCA II and hCA IV, but it is less effective as hCA I inhibitor, the triphenylpyridinium **2** is a quite effective hCA XII inhibitor, it also inhibits hCA IX, but its affinity for the other isoforms is in the micromolar range (see Table 1). Thus, sulfonamide **2** shows the most isoform-selective inhibition profile among the eight compounds considered here. Connected to the fact that it is a membrane-impermeant compound^36^^,^^37^, and that the presence of the three phenyl moieties may induce also a better lipophilic character compared to **1**, this compound constitutes an interesting lead for obtaining molecules to be investigated in detail for the selective inhibition of the tumour associated CA isoforms. The most interesting feature of **2** with respect to **1** is its reduced ability to inhibit the ubiquitous hCA II, although maintaining good inhibition constants against hCA IX and hCA XII. Indeed, due to the fact that hCA II is an ubiquitous, house-keeping isoform, its inhibition may be detrimental when the targeting of the tumour-associated isoforms CA IX and XII is envisaged, leading to many undesired side effects^2^. Thus, to get more insights into the molecular basis responsible for the reduced affinity for hCA II of compound **2** with respect to compound **1**, the X-ray crystal structure of the hCA II/**2** adduct was solved and compared with that previously reported of the hCA II/**1** complex^41^.

**Figure 2.**
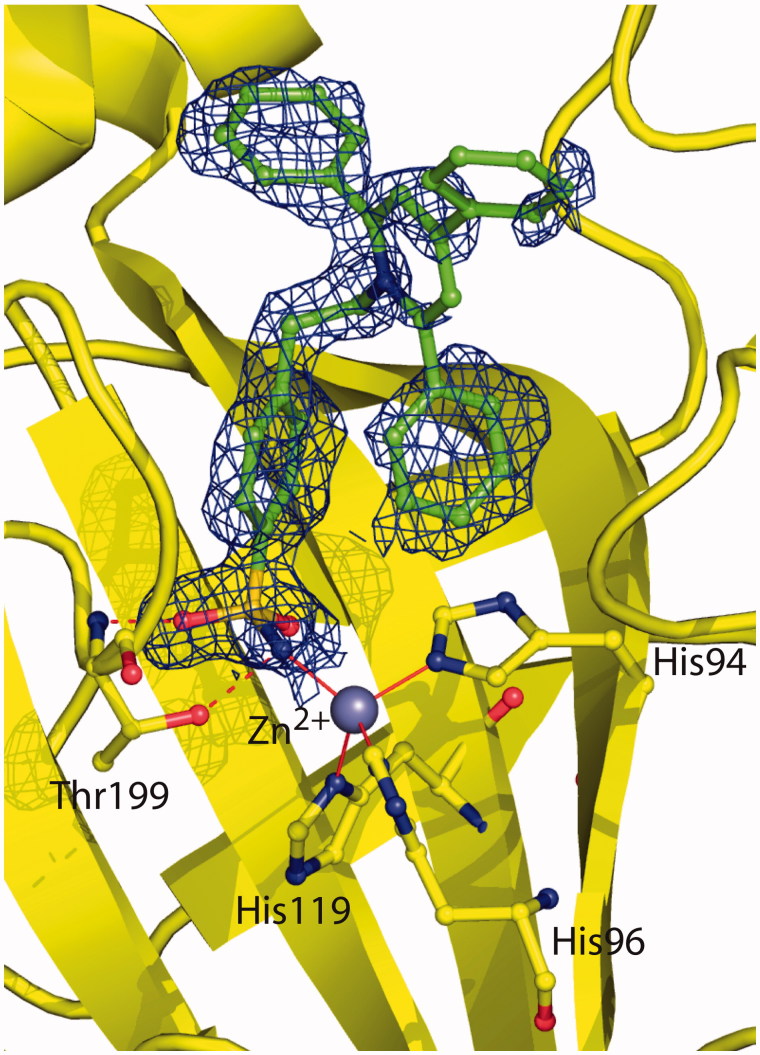
Active site region of the hCA II/**2** adduct, showing *σA*-weighted |2*F*_o_ − *F*_c_| simulated annealing omit map (contoured at 1.0*σ*) relative to the inhibitor molecule. Active site Zn^2+^ coordination (red continuous lines) and hydrogen bonds (red dotted lines) are also reported.

Crystals of the hCA II/**2** complex were obtained as previously described for other sulfonamide CA inhibitors^43^^,^^44^ and the structure was solved and refined using a previously reported procedure^46^^,^^52–55^. The final refined model had an *R*-work and *R*-free value of 17.5% and 21.0%, respectively, and was of high overall quality with 88.2% of the non-glycine residues located in the most favoured regions of the Ramachandran plot (Table 2). Since the initial stages of crystallographic refinement, electron density maps showed the presence of the inhibitor molecule bound within the enzyme active site. However, these maps were very well defined for the 4-ethylbenzenesulfonamide part of the inhibitor but poorly defined for the 2,4,6-triphenylpyridinium group, indicating that this region was flexible within the active site cavity (Figure 2). Accordingly, *B*-factor values of this region (33.9 Å^2^) were higher with respect to those observed for the 4-ethylbenzenesulfonamide moiety (11.7 Å^2^). The binding of the inhibitor within the active site did not cause significant changes in the enzyme structure as demonstrated by the low value of the RMSD calculated by superposing the Cα atoms in the adduct and the non-inhibited enzyme (0.3 Å).

As expected for a benzenesulfonamide inhibitor, compound **2** was bound to the enzyme with its sulfonamide group coordinated to the zinc ion in a tetrahedral geometry^2^. This group was also involved in two hydrogen bond interactions with residue Thr199, as already described for all hCA II/benzenesulfonamide adducts so far structurally characterized (Figure 2)^2^. No other polar interactions were observed between the enzyme and the inhibitor. Indeed, the ethylbenzene moiety was located in the middle of the active site establishing several hydrophobic interactions with Leu198, whereas the 2,4,6-triphenylpyridinium moiety did not establish strong interactions with enzyme (see above).

**Figure 3.**
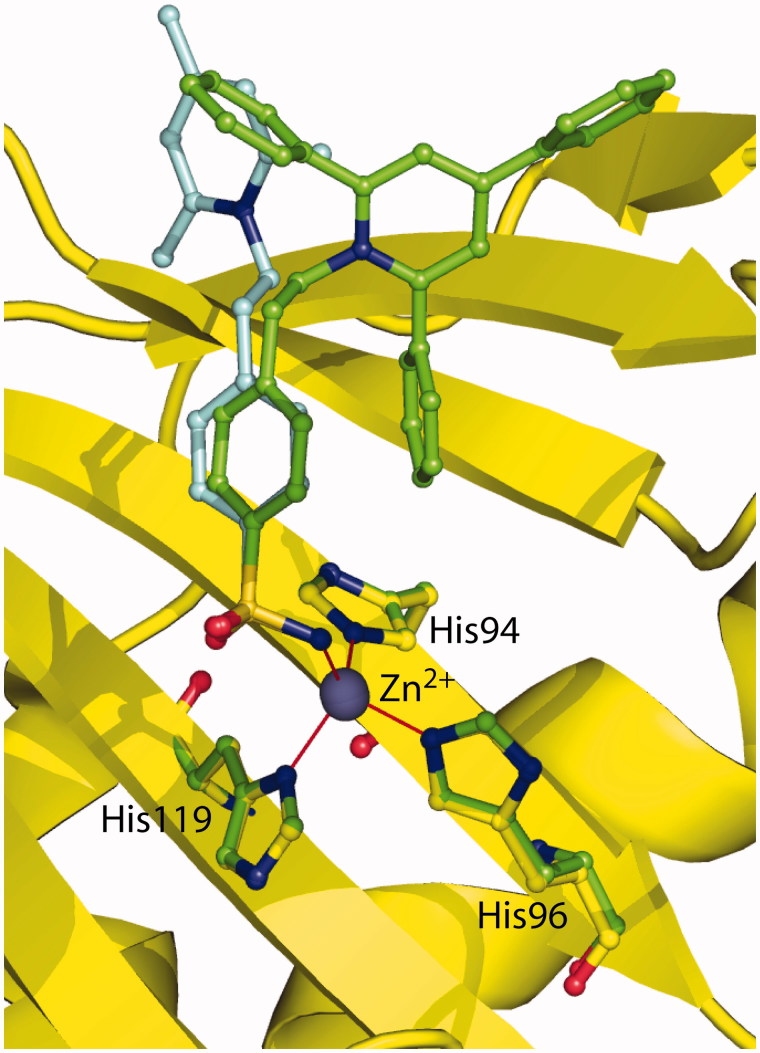
Structural superposition between **1** (cyan, PDB code 1ZE8)^41^ and **2** (green) when bound to hCA II active site.

**Figure 4.**
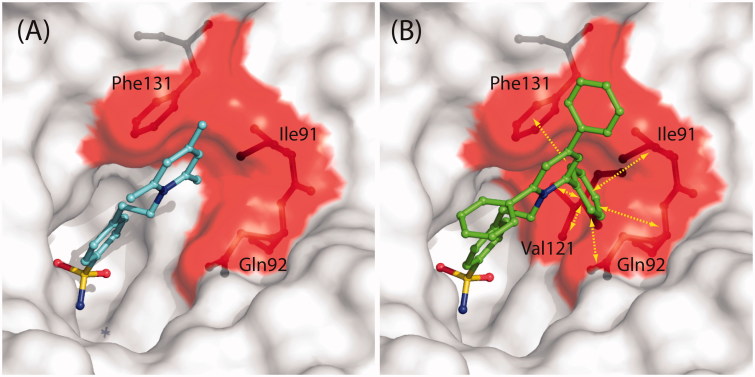
(A) Solvent accessible surface of hCA II/**1** active site^41^. Residues delimiting the hydrophobic pocket, where the trimethyl-pyridinium ring is located, are highlighted in red (Ile91, Gln92, Phe131). Compound **1** and residues Ile91, Gln92, and Phe131 are represented as ball-and-stick. (B) Solvent accessible surface of hCA II active site. Colour code is as in (A). The hypothetical conformation that compound **2** would have had if its pyridinium ring was positioned in the same hydrophobic pocket of **1** is shown. Yellow arrows indicate protein residues which clashes with inhibitor (Ile91, Gln92, Val121, Phe131).

Figure 3 reports the structural superposition of compounds **2** and **1** when bound to the hCA II active site, showing that even if the benzenesulfonamide groups of the two inhibitors are quite perfectly superimposable, the two trisubstituted pyridinium moieties are oriented toward different regions of the active site. In particular, the 2,4,6-trimethylpyridinium moiety of **1**, which was described as perfectly defined in the electron density maps^41^, fits perfectly into a hydrophobic pocket, defined by residues Ile91, Gln92, and Phe131, where it is involved in a strong face-to-face stacking interaction with the Phe131 aromatic ring (Figure 4(A)). On the contrary the 2,4,6-triphenylpyridinium moiety of **2**, although oriented toward the hydrophilic part of the active site, is flexible and does not establish many stabilizing interactions with enzyme’s residues. Interestingly, the only differences between the two inhibitors are the substituents of the pyridinium ring, namely three methyl groups for compound **1** and three phenyl moieties for compound **2**, which make it much more bulky and do not allow its accommodation into the hydrophobic pocket defined by residues Ile91, Gln92, and Phe131. Indeed, in this position the substituted ring would strongly clash with residues which delimit the pocket (Figure 4(B)). The impossibility for the pyridinium ring of **2** to be accommodated within the aforementioned hydrophobic pocket leads to the loss of the strong face-to-face interaction with Phe131 and is most likely responsible of its lower affinity for hCA II with respect to compound **1**.

## Conclusions

The X-ray data presented here explain why the triphenylpyridinium-substituted sulfonamide **2** is a much weaker hCA II inhibitor compared to its structural analogue **1** incorporating three methyl moieties at the pyridinium ring. Although there is a decreased affinity of **2** also towards the tumour-associated isoforms hCA IX and XII, compared to compound **1** (acting as a very potent inhibitor against both these isozymes), the triphenyl derivative **2** showed a selective inhibition profile for the tumour over cytosolic isoforms, which represents a valuable feature for compounds to be investigated as antitumor agents. Thus, understanding the detailed interactions between inhibitors belonging to similar structural classes, as **1** and **2** discussed here, may shed new light on the basic structural requirements for designing sulfonamide CAIs with a selective inhibitory profile.
